# Supplementary material for: DIA-Based Quantitative Proteomics in the Flower Buds of Two Malus sieversii (Ledeb.) M. Roem Subtypes at Different Overwintering Stages
Source: Int J Mol Sci. 2024 Mar 4;25(5):2964. doi: 10.3390/ijms25052964 (PMC10932172; doi:10.3390/ijms25052964)
Supplement: Supplementary file 1 [file ijms-25-02964-s001.zip › Supplementary Figure.pdf]

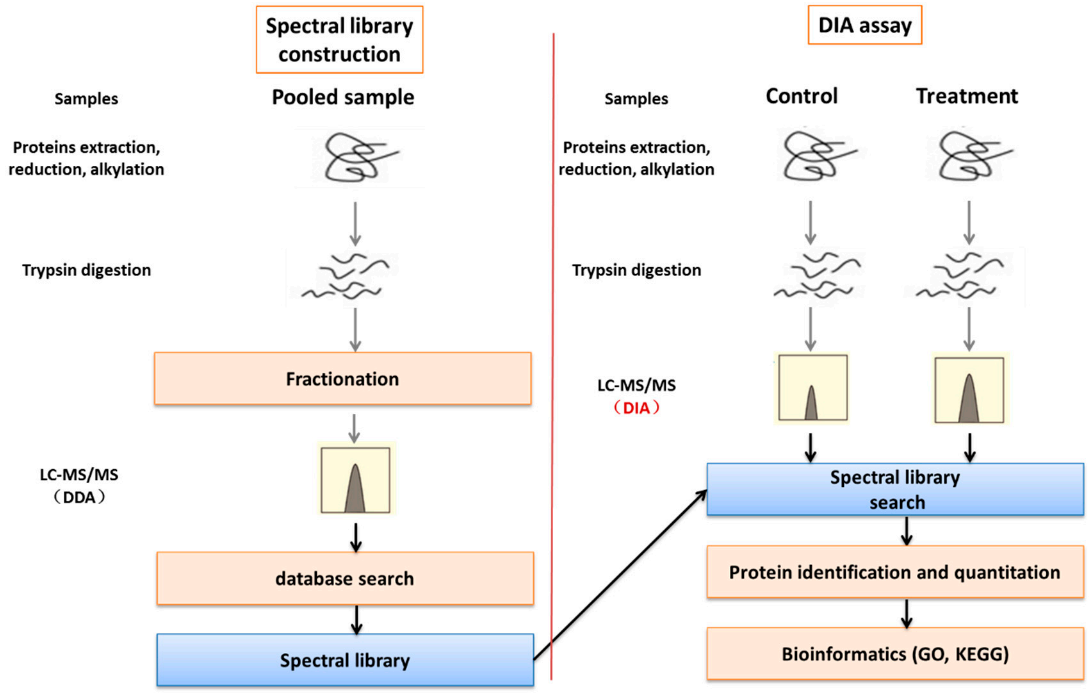


**Figure S1.** Flow chat of DIA-based on quantitative proteomic analysis in this experiment.

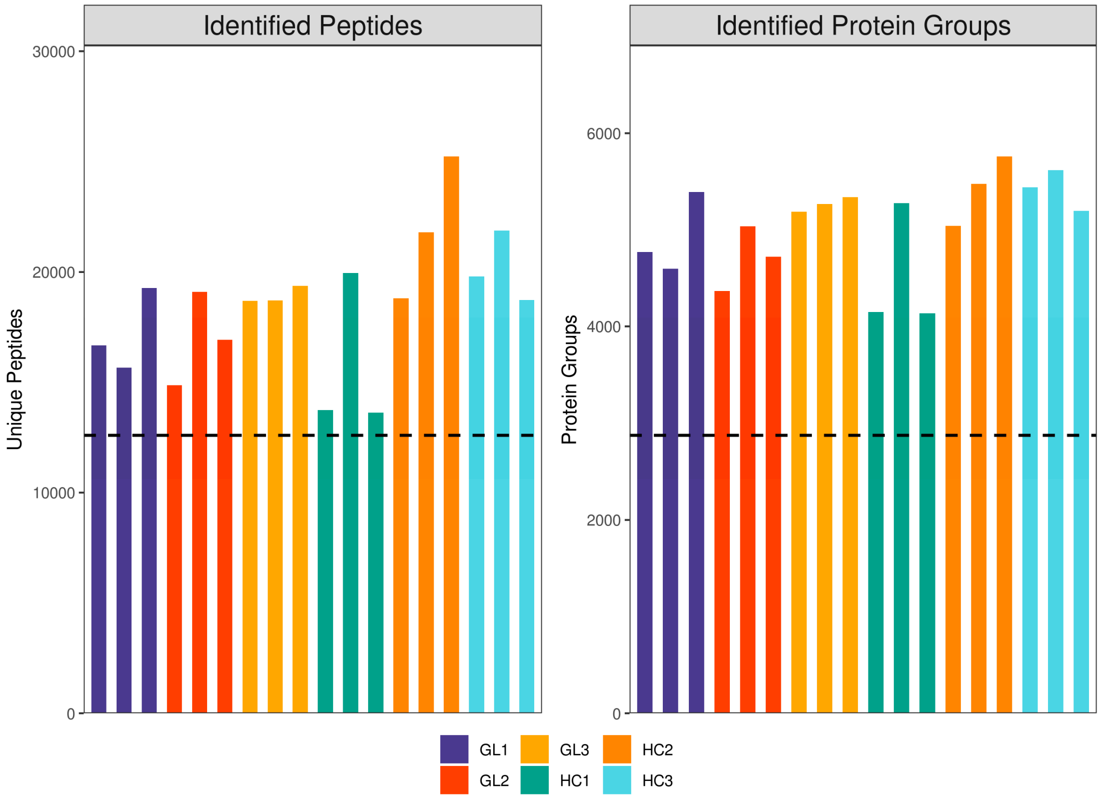


**Figure S2.** The number of peptides and proteins identified in each sample.

Note: Different colors represent different groups. The dashed lines represent the number of proteins or peptides at 50% of the total highest identified number.

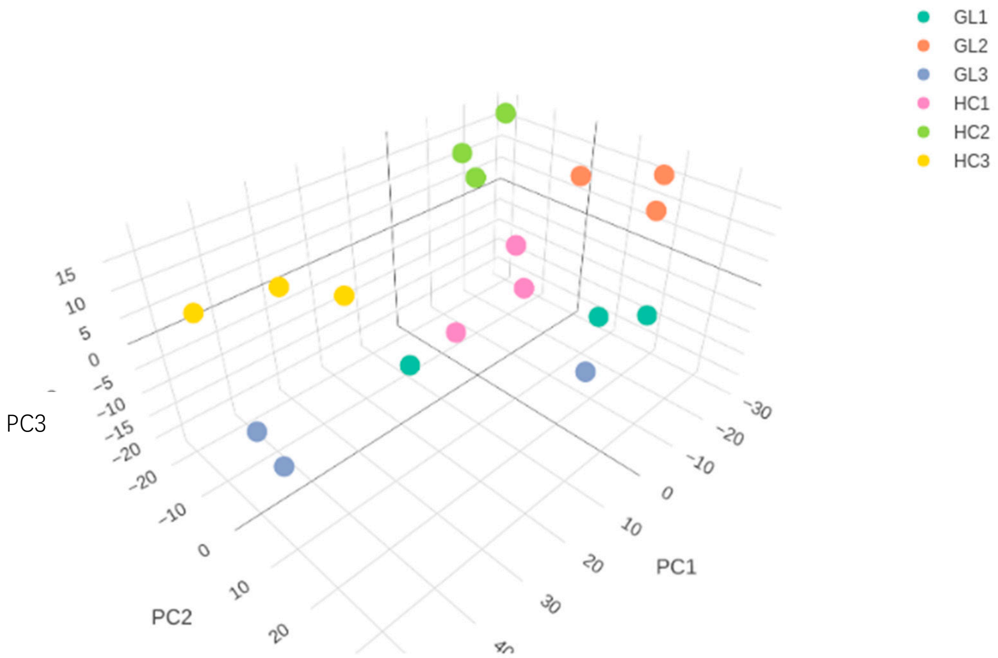


**Figure S3.** The principal component analysis (PCA) of identified proteins in each sample.

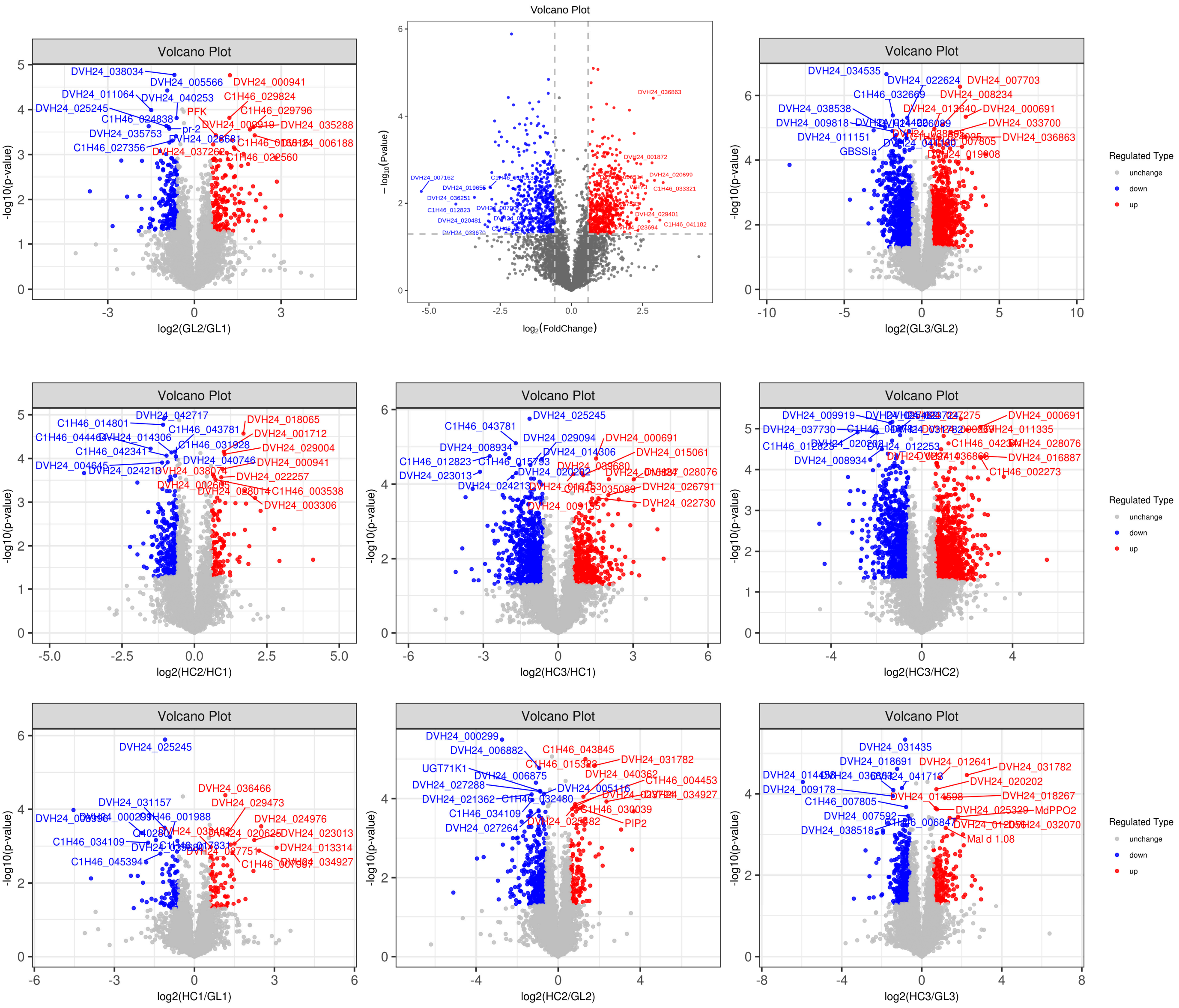


**Figure S4.** The volcano pictures of DEPs between comparison groups.

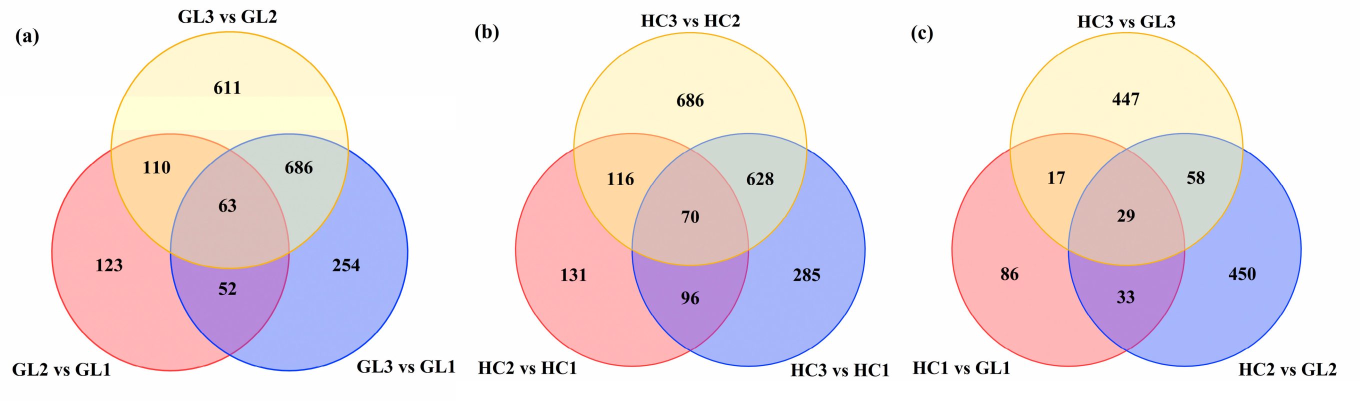


**Figure S5.** Venn diagram of DEPs between comparison groups base on the two subtypes and the overwintering stages.

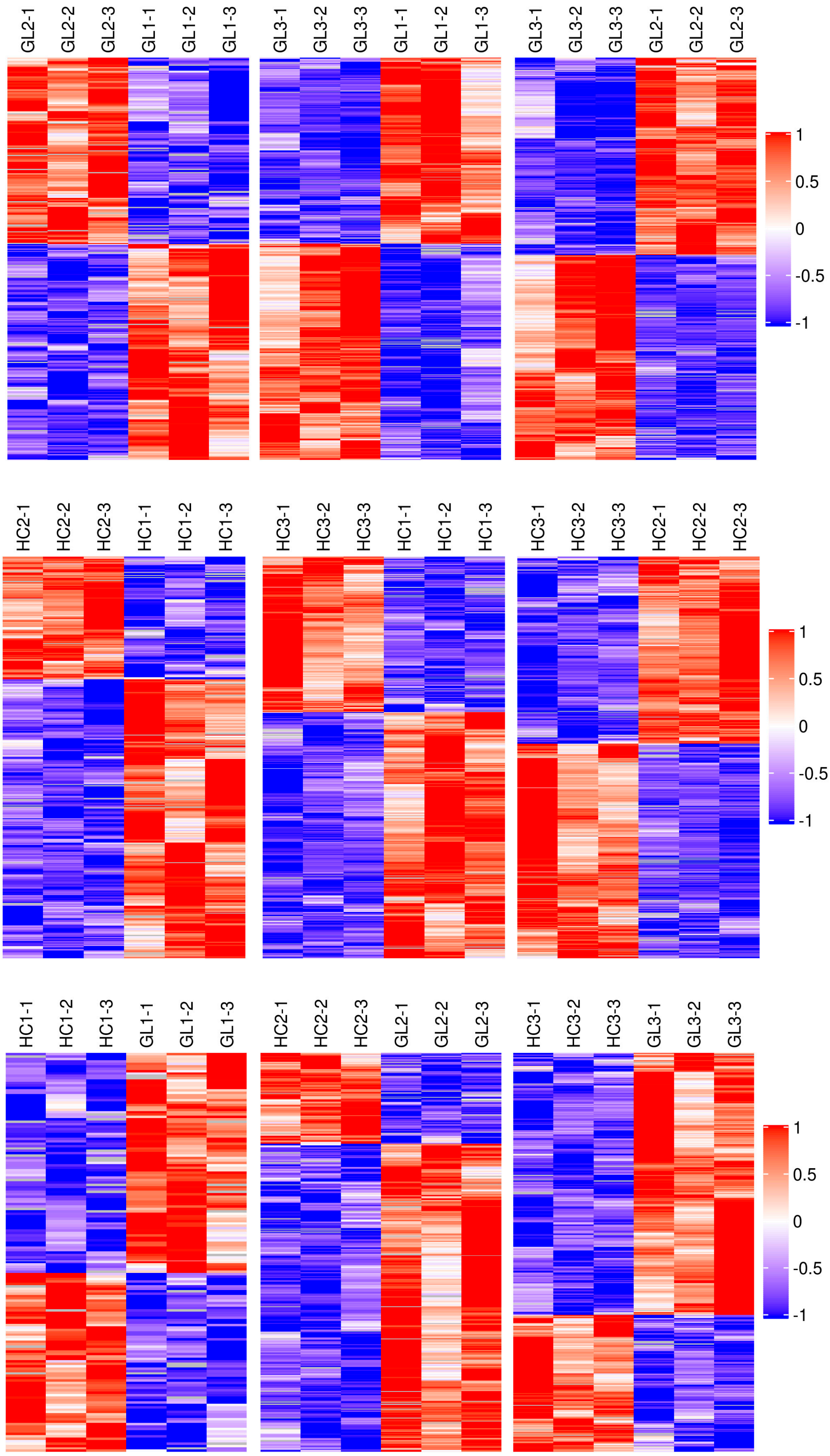


**Figure S6.** Cluster analysis of differentially expressed proteins between groups. The abscissa represents sample information, while the ordinate represents proteins with significant differential expression. Red represents significantly upregulated proteins, blue represents significantly downregulated proteins, and gray represents no protein quantitative information.

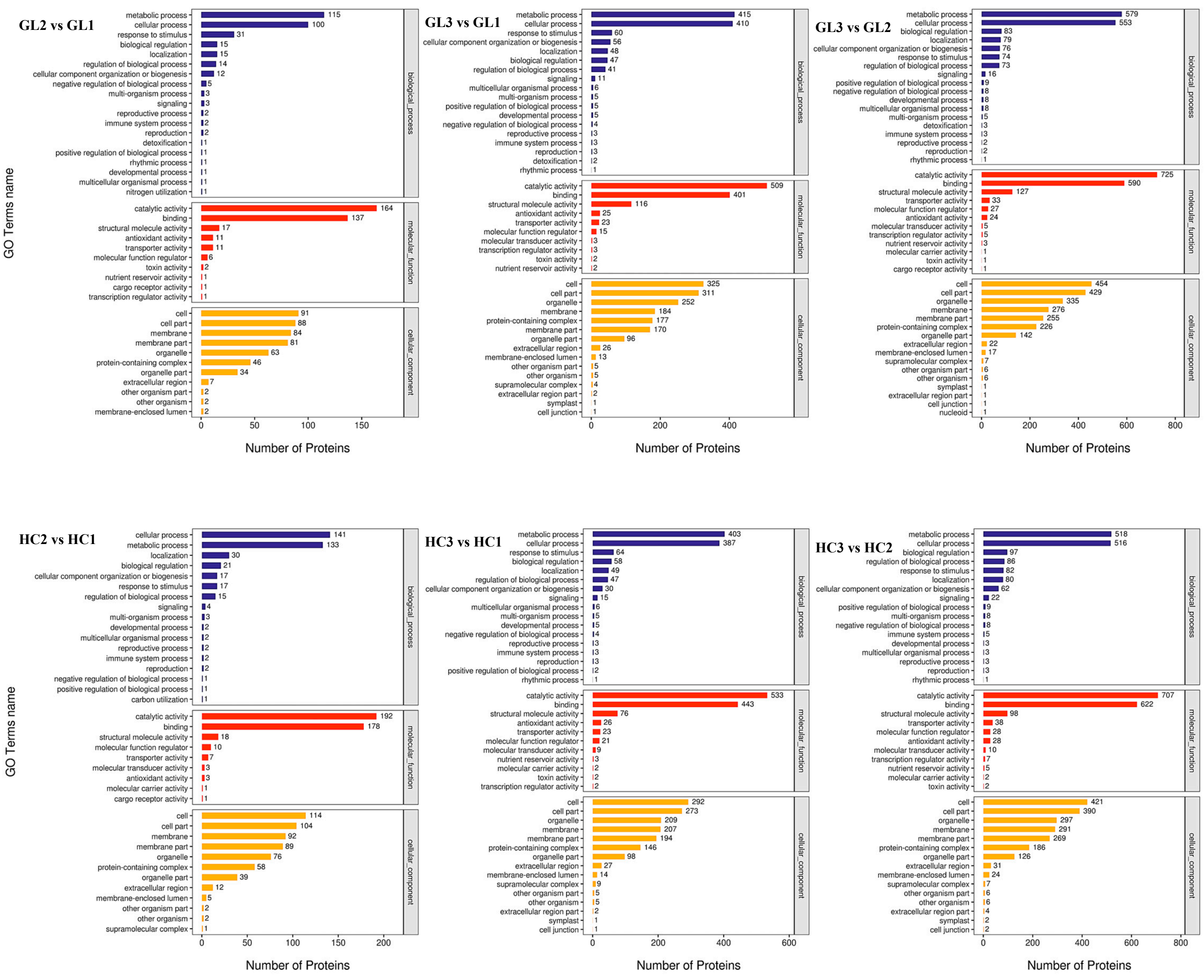


**Figure S7.** GO analysis of DEPs at different wintering stages in GL and HC, respectively.

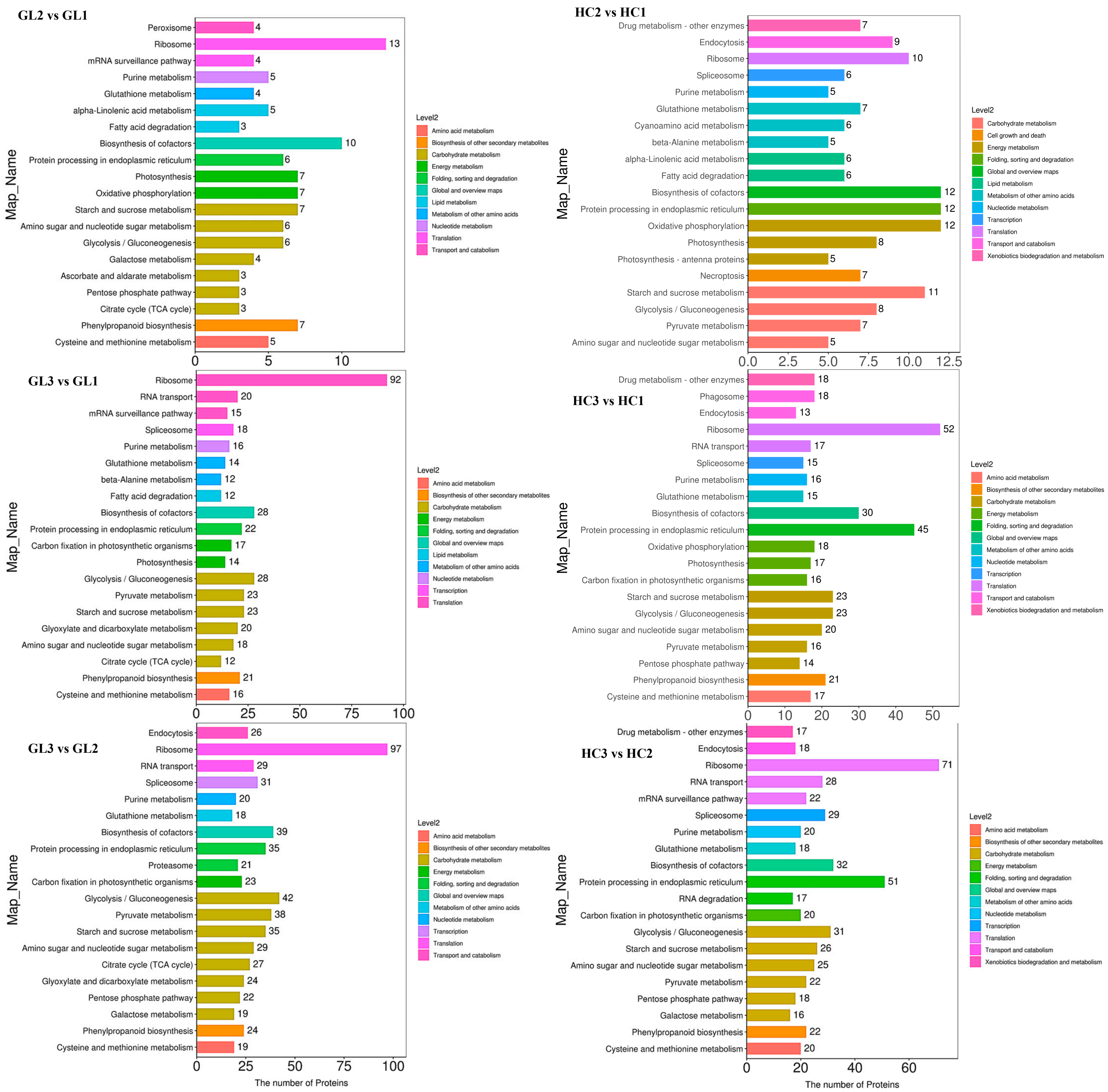


**Figure S8.** KEGG pathways of DEPs at different wintering stages in GL and HC, respectively.
